# The mandibular plane: a stable reference to localize the mandibular foramen, even during growth

**DOI:** 10.1007/s11282-019-00381-6

**Published:** 2019-04-03

**Authors:** David Feuerstein, Leonor Costa-Mendes, Rémi Esclassan, Mathieu Marty, Frédéric Vaysse, Emmanuelle Noirrit

**Affiliations:** 1 Department of Paediatric Dentistry, Dental Faculty, Paul Sabatier University and University Hospital of Toulouse, Toulouse, France; 2 Department of Oral Surgery, Dental Faculty, Paul Sabatier University and University Hospital of Toulouse, Toulouse, France; 3 Department of Prosthodontics, Dental Faculty, Paul Sabatier University and University Hospital of Toulouse, Toulouse, France; 4 Molecular Anthropology and Image Synthesis Laboratory (CNRS), UMR 5288 CNRS, Paul Sabatier University, Toulouse, France; 5 Department of Dental Medicine, Karolinska Institutet, Huddinge, Sweden

**Keywords:** Mandibular foramen, Mandibular plane, CBCT

## Abstract

**Objectives:**

The location of the mandibular foramen is essential for the quality of the inferior alveolar nerve block anaesthesia and has often been studied with contradictory results over the years. The aim of this study was to locate the mandibular foramen, according to the dental age of the subject, through 3D analysis.

**Methods:**

Three-dimensional images were reconstructed from mandibular computed tomography of 260 children, adolescents and adults. The occlusal plane was determined as the average plane passing through the buccal cusps of mandibular molars, premolars, and canines, and through the incisor edge. The mandibular foramen was located three dimensionally in relation to the anterior edge of the ramus (or coronoid notch), the sagittal plane and the occlusal plane.

**Results:**

All along mandibular growth, the three distances defining the relative position of the mandibular foramen showed negligible changes. The mandibular foramen is located from − 0.4 to 2.9 mm above the occlusal plane. The distance between the mandibular foramen and the leading edge of the mandibular ramus ranged from 17 to 19.5 mm. The angle between the ramus and the sagittal plane ranged from 3° to 5.4°.

**Conclusion:**

In our sample, and using the occlusal plane and the anterior edge of the ramus as anatomical references, the location of the mandibular foramen was considered to be similar in all patients regardless of age.

## Introduction

The inferior alveolar nerve block (IANB) is of considerable interest in dentistry and oral surgery. This procedure is also recommended by the American Association of Paediatric Dentistry [1]. For extraction of teeth in infected sites or endodontics, local infiltration may not be as effective as a mandibular block in molars [2]. Unfortunately, this technique has one of the highest failure incidences among dental anaesthetic techniques [3] with a reported failure rate between 15 and 30% [4, 5]. The reasons for failure include (1) the absence of indisputable anatomical landmarks [6]; (2) anatomical variations; and (3) poor anaesthetic technique [7, 8]. The position of the mandibular foramen (MF) in children is still debated and a lower level of the MF than in adults is considered as a standard in primary dentition [9]. The importance of locating the MF relative to anatomic structures that are convenient to use, regardless of the skill of the operator, is undeniable [10]. Many studies have tried to determine its position, but the results are often contradictory and the reference landmarks are sometimes difficult to identify clinically, especially in children [6, 11–14]. Previous attempts to determine the position of the MF relative to the occlusal plane have mainly been based on dry mandibles [7, 15, 16] or on 2D radiographs which were deformed (panoramic radiographs [6, 11–14, 17–19]) and/or had a high level of anatomical superimposition (lateral cephalometric radiographs [20, 21]). Nowadays, the development of 3D computer software combined with computed tomography (CT) allows indisputable data on the mandibular foramen location to be produced [10, 22–29]. The use of clinical reference landmarks should provide accurate measurements and convenient clinical transposition [10]. Therefore, in this study we aimed to determine the 3D position of the MF with respect to the occlusal plane (OP) and the mandibular ramus in groups with different states of dental maturation using a large sample of 3D images.

## Materials and methods

### CT-scanned sample

The data reported here involved no experimentation on human subjects, but only reprocessing of existing scan data. CT scans had been anonymized by the medical institution. The use of these data for the present purpose respected French bioethical laws and was carried out in accordance with the Declaration of Helsinki. Written consent was given by the patients for their information to be stored in the hospital database and used for research purposes. The “Comité Consultatif pour la Protection des Personnes dans la Recherche Biomédicale” (CCPPRB, French IRB) approved the use of these data for the present purpose.

This study included all the patients who had received a scanner examination for diagnosis or treatment planning at the dental service of T… University Hospital from 2007 to 2010, which means 272 subjects. Patients presenting dental agenesis, extracted teeth, decayed dental crowns, cranial and facial dysmorphias, facial or mandibular asymmetry, maxillofacial trauma, anamnesis of surgical procedure and/or artefact due to metallic parts (e.g., metallic dental crowns) or movement were excluded. All scans satisfying inclusion criteria were gathered without calculating the sample size with power analyses before the study design. The subjects were classified into seven groups according to their dental maturation status. We referred to Hellman’s dental stages, based on eruptive phases and dental occlusion [30] (Table 1).

**Table 1:** Groups 1–7 based on the Hellman’s classification of dental development

Group	Hellman’s stage	Characteristics
1	IIA	Completion of primary occlusion
2	IIC	Eruptive phase of permanent first molar or incisor
3	IIIA	Eruption of permanent first molar or incisor completed
4	IIIB	Exchange phase of lateral teeth
5	IIIC	Eruptive phase of permanent second molar
6	IVA	Eruption of permanent second molar completed
7	IV C et V A	Eruptive phase or eruption of permanent third molar completed

The human computed tomography scans were obtained from medical 16-row multidetector (MD) spiral CT scanner (Philips MX8000 IDT 16, Philips Medical Systems, Best, Netherlands). The parameters were set as follows: tube voltage 120 kV, tube current 330 mA, collimation (16 × 0.75), slice thickness 0.3–0.8 mm, field of view 150–250 mm, resolution level 512 × 512 pixels. Voxel size ranged from 0.23 to 0.49 mm, depending on the field of view.

### Landmarking

**Fig. 1:**
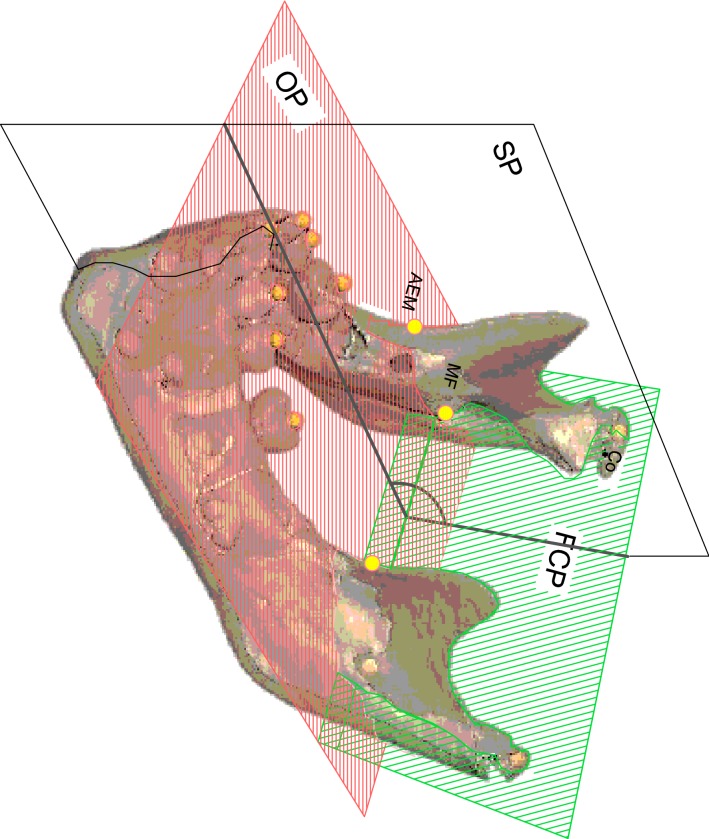
Localization of mandibular landmarks. The anterior edge of the mandibular ramus (AEM) was located and the distance between the MF and AEM was calculated

**Fig. 2:**
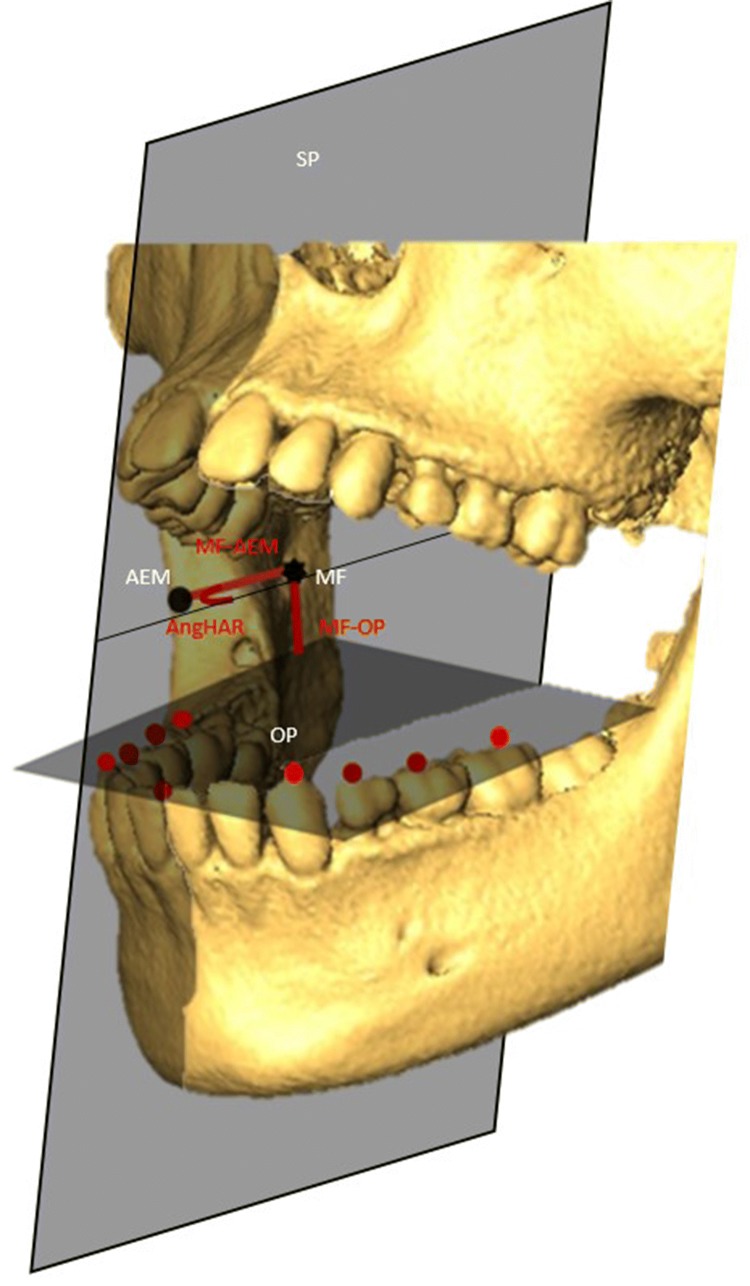
Construction of the average planes. The occlusal plane (OP) was modelized by computer using the landmarks located on the buccal cusps of the deciduous and/or permanent molars, premolars, canines and the inter-incisor point. The foramen condyle plane (FCP) was calculated by computer using the condyle landmarks and the mandibular foramen. The sagittal plane (SP) referred to landmarks located in the maxilla and the cranial base

We extracted from the CT data the Digital Imaging and Communications in Medicine (DICOM) file for each mandible and used Amira^®^ software (AMIRA, Visualization Sciences Group, Burlington, MA) for 3D reconstruction and measurement. To maximize the accuracy of the landmarking, points were manually positioned on 2D tomography and checked on 3D reconstruction (Table 2; Figs. 1, 2). These landmarks were translated into millimeter spatial coordinates (*x, y, z*) by the AMIRA^®^ software and used to measure distances and angles and to create planes. A plane is normally defined by three points. We decided for the first time to include at least four points per plane: all molar, premolar, canine and the inter-incisor point of the mandibular jaw in the occlusal plane (7–11 points instead of 3, to offer more stable values for statistical study), right and left condyles and mandibular foramina in the foramina condyle plane. These planes were modelized by computer using the least squares algorithm, meaning by a multiple regression calculation which made minimum the sum of the squares of the distances of each landmark projected orthogonally to an average plane. The least squares method is an analytical adjustment method according to which the function *f*(*x, y*) satisfies the equation: $S=\sum\nolimits_{{i=1}}^{n} {{{\left( {{z_i} - f\left( {{x_i},{y_i}} \right)} \right)}^2}}$. It allowed us to create an average plane out of a cloud of points. The average occlusal plane passing through the raised points (*x, y*_*i*_, *z*_*i*_) is the plane that gives the smallest distances of the points relative to this plane. The function *f* (*x, y*) is the equation of a plane of type *z* = *ax* + *by* + *c*. The plane sought is that which gives the minimum sum (*S*) in the equation $S=\sum\nolimits_{{i=1}}^{n} {{{\left( {{z_i} - (a{x_i}+b \cdot {x_i}+c)} \right)}^2}} ,$ where *a, b* and *c* are the parameters of the plane.

**Table 2:** Landmarks, distances, angle and planes used in the study

Mandibular foramen	Central point of the most upper part of the mandibular canal
rMF–lMF	Distance between the right and left mandibular foramina
Occlusal plane (OP)	Average plane passing through the buccal cusps of the mandibular molars, premolars and canines and the incisor edge
MF–OP	Orthogonal distance between MF and OP
Foramina condyle plane (FCP)	Average plane passing through the highest point of the right and left condyle upper surface and mandibular foramina
Ang OP/FCP	Angle between the OP and the FCP
Anterior edge of the mandibular ramus (AEM)	Most concave point of the anterior crest (coronoid notch) of the ramus
MF–AEM	Distance between MF and AEM
Sagittal plane (SP)	Average plane passing through the midsagittal points of the face and cranial base
Angle HAR (horizontal angle of the mandibular ramus)	Angle between the sagittal plane and the horizontal line passing through the MF and the AEM

50 subjects were measured twice on separate days (2 months interval) by one observer (DF), without knowledge of individual’s gender and age. Using the paired Student’s *t* test, the results showed no significant difference in all the measurements. Teeth with no occlusal relationship (under-positioned proximal contact points) on 2D and 3D reconstruction were excluded from the plane calculation.

### Statistical analysis

Statistical tests were performed using the R software package. Normality was tested using the Shapiro–Wilk test when *N* < 50 and d’Agostino tests when *N* > 50. Subjects that did not meet Grubbs’ test criteria between the first and the second measurements were excluded from the study. Measurement error (ME) was then tested using paired Student’s *t* tests. The correlation tests were performed to check if measurements were correlated with chronological age. Measurements were tested between Hellman’s stages, genders and sides using a one-way ANOVA and Tukey’s HSD post hoc tests corrected for multiple comparisons. The significance level was set at *p* < 0.05.

## Results

We initially screened 272 CT scans. After examination, 12 scans were excluded: 3 for tooth agenesis, 3 for decayed molar crown and 4 for syndromic dysmorphia. Two were excluded for not meeting Grubbs’ test criteria. The sample was finally composed of 260 CT scans, including 127 females and 133 males of mixed ethnicity from France, with a mean age of 12.38 ± 6.66 years (range 2.42–41.25 years) (Table 3). The distance between the two MF increased with age from 84 to 97 mm, particularly from G1 to G3. The growth speed decreased dramatically from G3 to G6 (no significant difference between these groups). Positive correlations were found between chronological age and the distance between the mandibular foramina (*r*^2^ = 0.284). No significant differences were observed between males and females and left–right symmetry was respected (Table 4). The two sets of measurements did not differ significantly (*p* = 0.769), thus providing an indication of the reliability of the method.

**Table 3:** Number of subjects according to Hellman’s dental maturation stages, ages, distances and angles [mean (SD)]

Hellman’s stages	(*n*) Per group	(*n*) Males/(*n*) females	Age	MF–OP	AngHAR	MF–AEM	rMF–lMF	AngOP
G1	30	16/14	4.1 (0.9)	− 0.4 (2.7)	4.0 (2.7)	17.0 (1.4)	84.4 (3.8)	120.8 (6.6)
G2	24	12/12	6.3 (1.0)	2.5 (2.9)	3.0 (2.8)	17.8 (1.7)	90.1 (3.3)	116.1 (4.8)
G3	37	20/17	9.6 (1.2)	0.1 (2.8)	3.5 (2.7)	18.2 (2.5)	92.1 (4.8)	120.4 (5.0)
G4	31	16 /15	10.8 (0.8)	0.3 (2.7)	3.3 (2.5)	18.4 (2.4)	91.7 (5.2)	119.6 (4.2)
G5	38	20/18	11.6 (0.8)	2.8 (3.0)	4.0 (3.2)	19.5 (2.4)	93.6 (4.9)	117.2 (5.1)
G6	50	23/27	12.8 (0.7)	0.4 (2.6)	3.9 (2.6)	18.8 (2.2)	93.8 (4.8)	120.3 (6.11)
G7	50	26/24	23.5 (6.1)	2.9 (3.3)	5.4 (4.0)	18.7 (2.3)	97.6 (5.1)	117.4 (9.3)

**Table 4:** Student’s and Fisher’s test results

	Fisher’s test		Student’s test	
	*F*	*p* value	*F*	*p* value
Boys and girls	0.875	0.125	− 0.417	0.677
Right and left	0.889	0.182	− 0.651	0.515
Measures 1 and 2	0.985	0.863	0.769	0.769

**Table 5:**
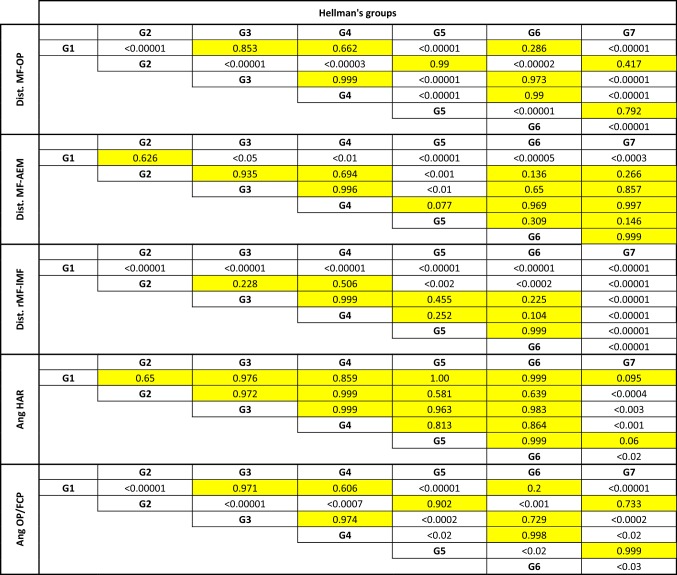
Tukey test results (painted boxes representing the non-significant difference)

**Fig. 3:**
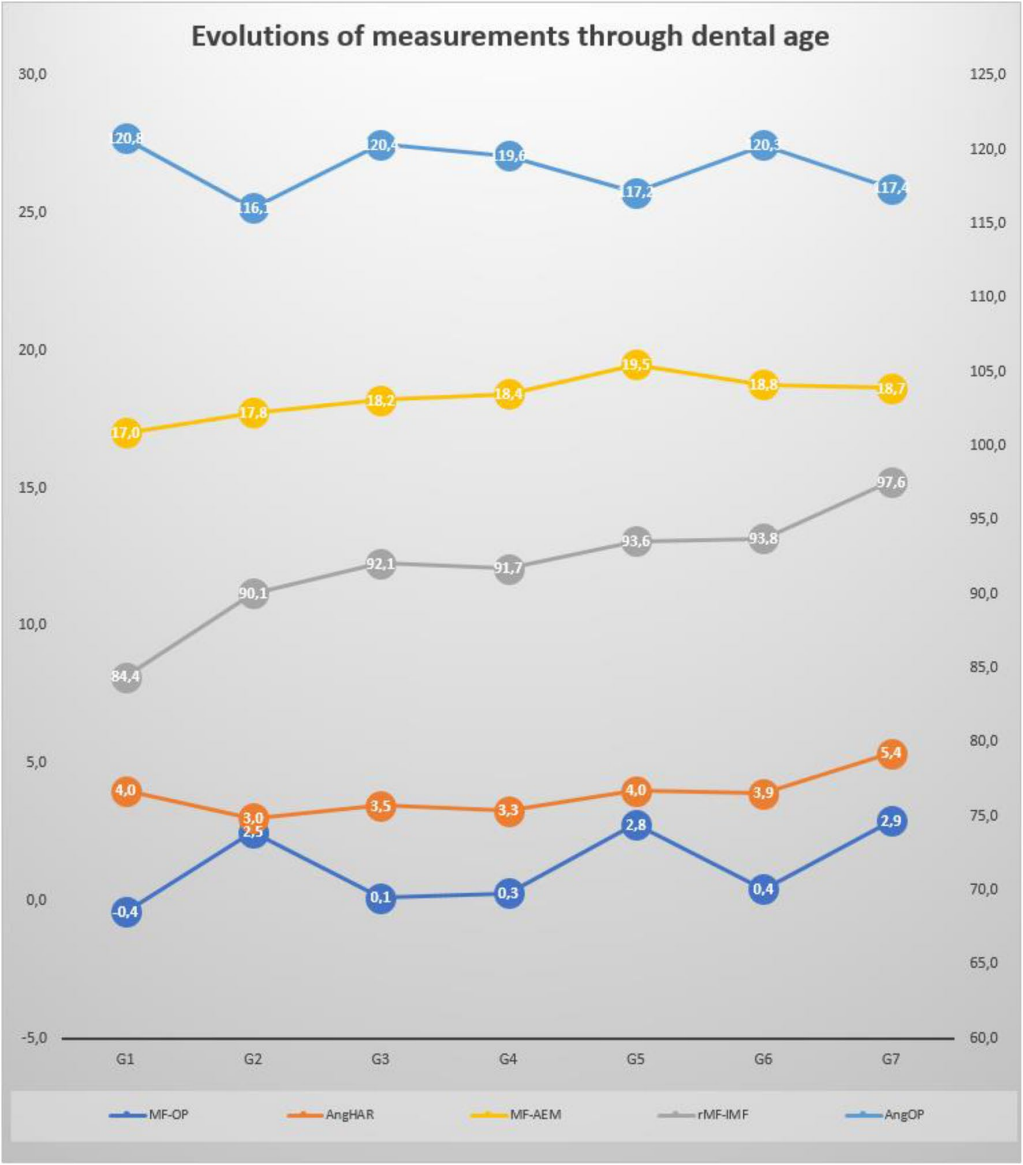
Evolutions of the measurements through dental age. *MF–OP* distance (mm) from the mandibular foramen to the OP, *MF–AEM* distance between the mandibular foramen and the most concave point of the anterior edge of the mandibular ramus (coronoid notch), *rMF–lMF* distance (mm) between the right mandibular foramen and the left mandibular foramen, *AngHAR (°)* angle between the sagittal plane SP and the line through the points MF and AEM, *AngOP (°)* angle between the OP and the FCP

**Fig. 4:**
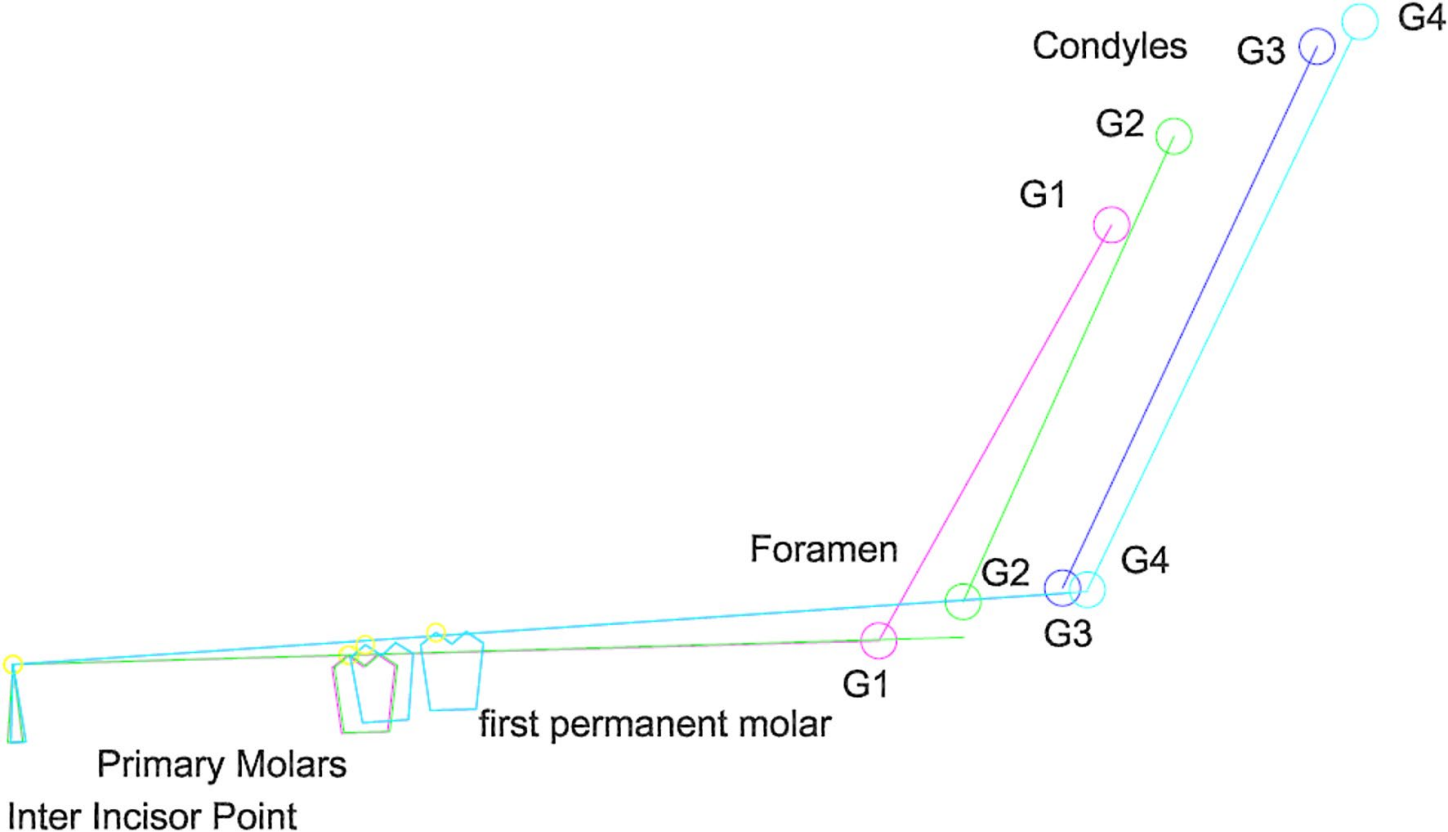
Growth diagram from G1 to G4

**Fig. 5:**
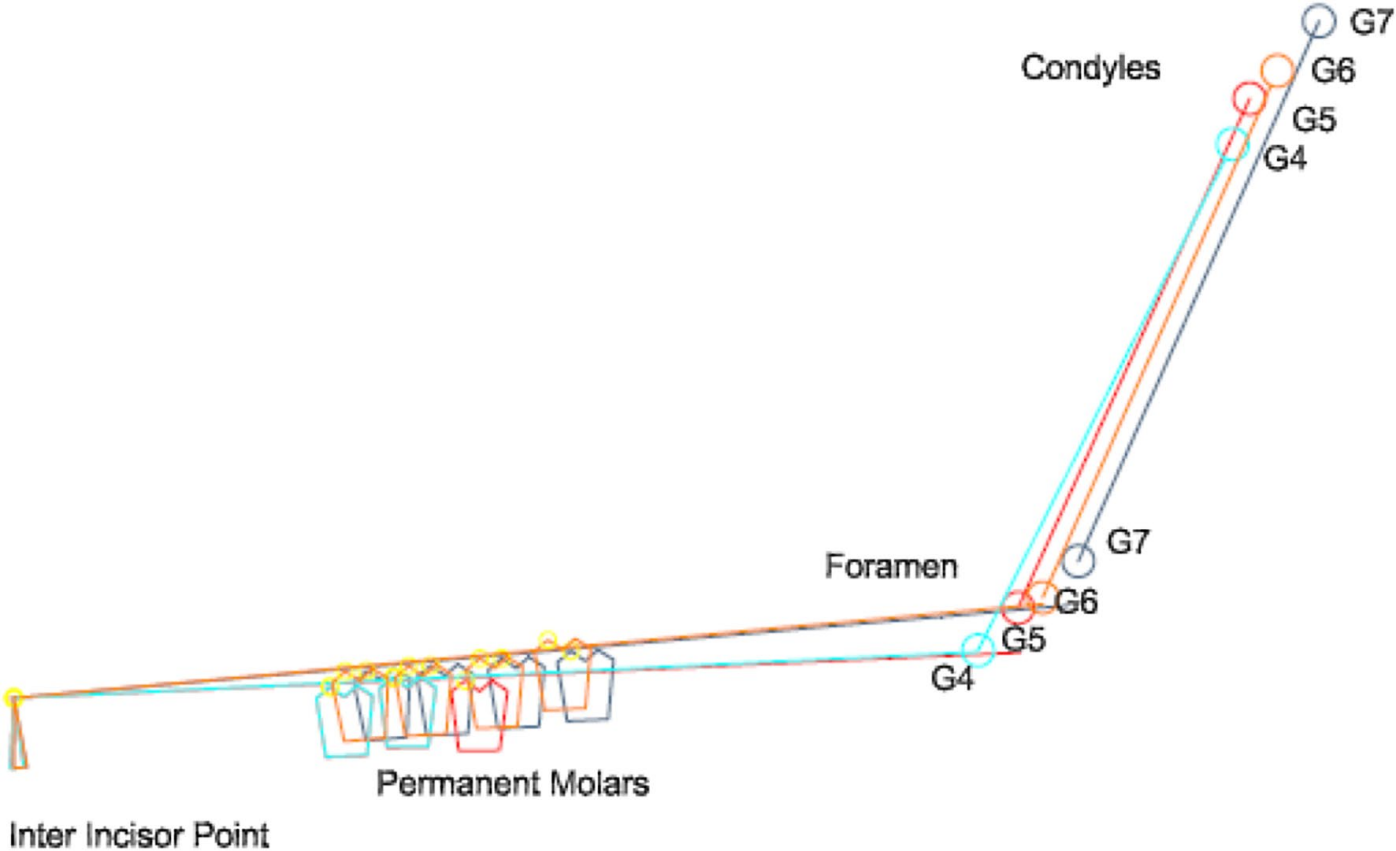
Growth diagram from G4 to G7

The average MF–OP (mandibular foramen–occlusal plane) distance was 1.31 ± 3.2 mm and varied little with dental age (Fig. 3). For G2 (6 years old), G5 (11.5 years old) and G7 (23 years old), the average distance varied from 2.5 to 2.9 mm above the occlusal plane. For G1 (4 years old), G3 (9.5 years old), G4 (10.5 years old) and G6 (13 years old), the average distance was practically zero (between − 0.4 and + 0.4 mm). The variations were especially large during the eruption of the permanent molars (first and second permanent molars) and stable otherwise, as was the angle AngOP/FCP (between the occlusal plane and the foramina condyle plane) (Figs. 4, 5). The angle AngOP/FCP varied between 116.1° and 120.8°. According to Hellman’s groups, the distance MF–OP and the angle AngOP/FCP showed significant differences between the three groups G2, G5, G7 and the other four groups (G1: *p* < 0.00001, G3 *p* < 0.0002, G4 *p* < 0.02, G6 *p* < 0.03) (Table 5).

The distance MF–AEM (anterior edge of the mandibular ramus) ranged from 17 to 19.5 mm (SD = 2.5 mm, 95% CI = 0.61). It increased slowly from G1 to G5 and remained stable after G5 (11.5 years). Only G1 and G5 showed significant differences with the other groups (*p* < 0.05). The angle HAR varied very little with age, from 3° to 5.4° (3.64° ± 0.42). Only the adult group (G7) was significantly different from the others (*p* < 0.02). According to the low intergroup variation of the previous results, no correlation was found between chronological age and the distances MF–OP (*r*^2^ = 0.04), MF–AEM (*r*^2^ = 0.019) or the angle HAR (*r*^2^ = 0.05).

## Discussion

To achieve inferior alveolar nerve block, it is essential to identify the position of the mandibular foramen (MF) in a simple and reproducible way, regardless of the patient’s age. That is why we used for the first time a modelized occlusal plane including all the erupted teeth as a reference in our measurements. We reported a stable position of the mandibular foramen (MF) related to the occlusal plane, the anterior edge of the ramus and the sagittal orientation of the ramus during growth.

Many studies have shown some differences in the location of the mandibular foramen, especially in terms of age [17, 20, 27]. Classifying subjects according to Hellman’s groups seemed to be more appropriate, taking into consideration the clinical situation of the mandibular growth instead of the chronological age. The transverse mandibular growth was observed by the inter-foramen distance (rMF–lMF), which increased continuously with age (G1 to G7 = 84–98 mm). Unlike the Epars study [21], we did not follow the patients over growth, but used a cross-sectional study of groups of population with different states of dental maturation, as did Kang [10]. Therefore it cannot be formally concluded that the mandibular foramen may stay at the same level from the occlusal plane when the patients get older, which can be considered as a limitation of our study.

In agreement with most previous studies [6, 7, 13, 26, 29], no significant differences were observed between boys and girls. Movahhed and Kanno found a different location of the foramen with sex, but only at the age of 9 years, probably due to the difference in mandibular growth at this age [11, 19]. Park [26] established a significant difference in the position of MF between adult men and women in skeletal class III malocclusion.

No significant differences between the measurements on the right and left were observed as in other studies [14, 19, 22, 26, 29]. Facial symmetry was respected. This confirmed that the subjects studied were anatomically normal without mandibular growth deviations.

Studies about the localization of the MF often referred to different methods, media and landmarks. Palti [15], Monnazzi [16] and Thangavelu [7] performed studies on dry mandibles and found that the foramen was located close to the occlusal plane, as we did. Nevertheless, while the analysis of dry mandibles could locate anatomical landmarks precisely, the location of imaginary lines and subjective constructions, such as the occlusal plane, might be difficult. Consequently, the resulting measurements became less accurate. Moreover, the sex and age of the mandibles were unknown and the small number of subjects, compared to other methods, reduced the statistical power. Two studies based on panoramic radiographs and measurements on the bony ridge [12, 13] found that the MF position did not significantly change with age. However, even if the alveolar crest location appeared more convenient in panoramic radiographs, it critically lacked clinical relevance as it was masked by the oral mucosa. In addition, the exact location of the mandibular foramen on radiographs, described as the most superior anterior point of the mandibular canal [19], or based on the triangular opacity of the lingula [17] or a point set postero-superiorly to the opening of the mandibular canal [26] is not always easy to establish due to its radiolucency [16]. For studies using cephalometric radiographs, the MF was first located on panoramic before being transferred on the cephalometric radiograph to overcome the superimposition of contralateral mandibular structures [21]. The occlusal plane was defined on panoramic radiographs as a hand-drawn line passing through the cusps of the molars. Movahhed used a software to draw it and calculate linear measurements [19]. He reported different results according to sex and age: at age 9 years, the mandibular foramen remained below the occlusal plane in boys, while in girls it was above it. It may be explained by the different patterns of growth between boys and girls. Moreover, its reference line passed through the canine tip and “the most prominent point on the end-most fully erupted tooth” which differed from our calculated plane involving all the dentition.

Skeletal malocclusions have been shown to impact the distance between MF and OP. In young adult CBCT, Park [26] described an MF close to the OP in the normal and skeletal class II occlusion group, and a distance of 2.8 mm above the OP for skeletal class III malocclusion group. In a study on cephalometric and panoramic radiographs of patients who underwent orthodontic treatment, Epars [20, 21] found that this distance increased with growth and to a greater extent for short face individuals compared to long-face individuals, because of the mandibular rotation. But this evolution could be also influenced by orthodontic treatment. According to Epars, the MF located below the occlusal plane level in younger patients gradually rose to be above it in older patients, reaching the occlusal plane level by the age of 8.5 years. On average, the mandibular foramen was 3.1 ± 2.6 mm above the occlusal plane, which is consistent with our results. In the same way, in a population of children with Mongoloid skeletal pattern, Lim [18] described a higher percentage of MF located below the OP under 9 years, and above in older children. In line with our results, Paryab [17] confirmed this position of MF 3–4 mm above the mandibular plane in mixed dentition (from Hellman’s stages IIA to IVA), whether the patients were retrognathic or not.

Six other studies have analyzed the mandibular lingula (ML) position on panoramic radiographs [6, 11, 14, 19] or on CBCT [24, 25]. The ML is a tongue-shaped bony projection on the medial surface of the mandibular ramus [24], situated superiorly and anteriorly to the mandibular foramen [16]. Kanno found that the lingula was above the OP in 70% of girls and 55% of boys in the 7  years old group and in 85% of all children by age 10 [11]. Krishnamurthy described the location of the ML of 2–3 mm above the occlusal plane in 7 and 8 years old children, increasing until 6 mm for children of the ages 11–12 years [14]. In Sekerci study [24] using CBCT, the authors reported a mean height level of the mandibular lingula of 2 mm above the occlusal plane. On the contrary, Ezoddini found on panoramics that the lingula was 0.5 mm below the OP [6].

Finally, the differences with our study could be explained in four different ways: (1) the mandibular lingula was located above the MF, with great variation in the ML–MF distance [16, 28] and its anatomical forms [25]; (2) panoramic radiographs might be subject to deformations [31] and the distances measured on dry mandibles and panoramic radiographs have showed significant differences [32]; (3) in three of these studies, the OP was drawn by hand; (4) ethnic anatomical differences (Korean, Brazilian, Iranian, Indian populations) could exist due to ancestral dissimilarity [27].

Thanks to advances in CT scanning and 3D reconstruction software, the situation of MF can be nowadays more accurately studied, without distortion of the anatomical regions. We reported a stable vertical location of the mandibular foramen, 2–3 mm above the occlusal plane in adults and in children during the eruption of the permanent molars and at the same level as the occlusal plane in between. Our results are in line with other studies, except with one conducted in a young adult population: Zhou [28] reported a mandibular lingual located above the occlusal plane, but an MF below OP in 84% of the cases. The results of the Korean study of Kang [10] are slightly higher than those of ours, but the definition of the occlusal plane by only three landmarks located in the permanent teeth, as in Zhou study [28], might be less representative of the clinical reality than our adjusted occlusal plane. Nevertheless, MF was located at a stable distance of 3.2–3.8 mm above the occlusal plane, both in the adult and the growth groups [10]. Altunsoy [29] found that the MF was 2.5–3.6 mm above the occlusal plane of the molars, without statistically significant changes with age. More markedly, Blacher [23] reported a location of MF 9.8 mm above OP in adults while using an occlusal plane passing only through the second mandibular molar and the MF. Al-Shayyab reported an average distance of 4.5 mm between the MF and OP in adults [23]. In this CBCT study, the occlusal plane was represented by the straight line of the cusps of the mandibular first molar, extended posteriorly by the software ruler. The average occlusal plane of our study might offer statistically steadier values.

As the mandibular foramen seemed to follow the vertical evolution of the occlusal plane with molar eruption, the sagittal angulation of the ramus, defined by the angle between OP and FCP (plane defined by both right and left MF and condyles), stayed stable.

Sagittally, the rearward movement of the foramen relative to the anterior edge of the mandible has also been described in some studies [10, 27]. However, this displacement remained very small with an average distance of 18.4 ± 2.3 mm, confirmed by the CBCT measures of Park (19.4 mm ± 2.1) [26] and Zhou (18.3 ± 2.2 mm) [28], but not by those of Findik [22] (around 15.3 mm in the adult group and children group).

All these measurements allowed us to conclude that clinicians could rely on constant anatomic references to identify the position of the mandibular foramen when performing IANB both in children and adults. In our sample, the mean distance between the MF and the average OP was 1.3 mm. The occlusal plane could then be a useful reference for the insertion height of the injection needle in adults and paediatric patients, as our sample did not exhibit significant differences between the dental age groups. A slight correlation was found between dental age and the MF–AEM distance with an average of 18.4 mm. This value could also be a reference for the insertion depth of the needle regardless of age. The horizontal angulation of the ramus, measured by the angle HAR, ranged between 3° and 5.4° and did not show significant variation in children and adolescents (G1 to G6). Finally, to achieve the mandibular nerve block, we suggest 1) to visualize the occlusal plane virtually or by positioning the thumb along the cusps of the erupted teeth; 2) to insert the needle 5 mm above the occlusal plane with a penetration depth around 19 mm from the anterior edge of the mandibular ramus and an angulation of 45° in all patients regardless of age.

## Conclusions

Dental landmarks are often neglected when anaesthesia is performed because many older studies showed a significant change in the foramen location relative to the teeth, during mandibular growth. This original 3D study contributes new information about the position of the mandibular foramen in growing patients. It has shown that the foramen moved slightly backward and upward during mandibular growth and the occlusal plane followed this development with the eruption of the first and second permanent molars. Thus, the dental arch can be used as a reference plane for performing a mandibular nerve block.
